# Supplementary material for: Psychometric Properties of the Berger HIV Stigma Scale: A Systematic Review
Source: Int J Environ Res Public Health. 2021 Dec 11;18(24):13074. doi: 10.3390/ijerph182413074 (PMC8701211; doi:10.3390/ijerph182413074)
Supplement: Supplementary file 1 [file ijerph-18-13074-s001.zip › ijerph-1482569-supplementary/Supplementary File S1.pdf]

Supplementary File S1

(((((((((((((((((((((((("social stigma"[All Fields] OR "stereotyp*"[All Fields]) OR (("prejudice"[MeSH Terms] OR "prejudice"[All Fields]) OR "prejudices"[All Fields])) OR "discriminat*"[All Fields]) OR "social perception"[All Fields]) OR (((((("shame"[MeSH Terms] OR "shame"[All Fields]) OR "shames"[All Fields]) OR "shaming"[All Fields]) OR "shamed"[All Fields]) OR "shameful"[All Fields]) OR "shamefulness"[All Fields])) OR (("guilt"[MeSH Terms] OR "guilt"[All Fields]) OR "guilts"[All Fields])) OR "self blame"[All Fields]) OR "self criticism"[All Fields]) OR ((((("social stigma"[MeSH Terms] OR ("social"[All Fields] AND "stigma"[All Fields])) OR "social stigma"[All Fields]) OR "stigma"[All Fields]) OR "stigmas"[All Fields]) OR "stigma s"[All Fields])) OR "social marginalization"[All Fields]) OR ("fear"[MeSH Terms] OR "fear"[All Fields])) OR "social distanc*"[All Fields]) OR "social exclusion"[All Fields]) OR "self concept"[All Fields]) OR "self perception"[All Fields]) OR "ostrac*"[All Fields]) OR (("taboo"[MeSH Terms] OR "taboo"[All Fields]) OR "taboos"[All Fields])) OR "social conformity"[All Fields]) OR "marginal*"[All Fields]) OR "social discrimination"[All Fields]) OR "vulnerable populations"[All Fields]) OR "social acceptance"[All Fields]) OR "social alienation"[All Fields]) AND ((((((("human immunodeficiency virus"[All Fields] OR "acquired immunodeficiency syndrome"[All Fields]) OR "hiv serodiagnosis"[All Fields]) OR ((("acquired immunodeficiency syndrome"[MeSH Terms] OR (("acquired"[All Fields] AND "immunodeficiency"[All Fields]) AND "syndrome"[All Fields])) OR "acquired immunodeficiency syndrome"[All Fields]) OR "aids"[All Fields])) OR "hiv infection"[All Fields]) OR ("hiv"[MeSH Terms] OR "hiv"[All Fields])) OR (("curr opin hiv aids"[Journal] OR ("hiv"[All Fields] AND "aids"[All Fields])) OR "hiv aids"[All Fields])) OR ((("acquired immunodeficiency syndrome"[MeSH Terms] OR (("acquired"[All Fields] AND "immunodeficiency"[All Fields]) AND "syndrome"[All Fields])) OR "acquired immunodeficiency syndrome"[All Fields]) OR "aids"[All Fields]))) AND ((((((((((((("scale s"[All Fields] OR "scaled"[All Fields]) OR "scaling"[All Fields]) OR "scalings"[All Fields]) OR "weights and measures"[MeSH Terms]) OR ("weights"[All Fields] AND "measures"[All Fields])) OR "weights and measures"[All Fields]) OR "scale"[All Fields]) OR "scales"[All Fields]) OR "measure*"[All Fields]) OR "assess*"[All Fields]) OR (((((("instrument"[All Fields] OR "instrument s"[All Fields]) OR "instrumentation"[MeSH Subheading]) OR "instrumentation"[All Fields]) OR "instruments"[All Fields]) OR "instrumented"[All Fields]) OR "instrumenting"[All Fields])) OR "question*"[All Fields]) OR (((((((((("inventoried"[All Fields] OR "inventory s"[All Fields]) OR "inventorying"[All Fields]) OR "personality inventory"[MeSH Terms]) OR ("personality"[All Fields] AND "inventory"[All Fields])) OR "personality inventory"[All Fields]) OR "inventories"[All Fields]) OR "equipment and supplies"[MeSH Terms]) OR ("equipment"[All Fields] AND "supplies"[All Fields])) OR "equipment and supplies"[All Fields]) OR "inventory"[All Fields])) OR "tool"[All Fields])) AND ("berger"[All Fields] OR "berger's"[All Fields])
