# Supplementary material for: Psychometric Properties of the Berger HIV Stigma Scale: A Systematic Review
Source: Int J Environ Res Public Health. 2021 Dec 11;18(24):13074. doi: 10.3390/ijerph182413074 (PMC8701211; doi:10.3390/ijerph182413074)
Supplement: Supplementary file 1 [file ijerph-18-13074-s001.zip › ijerph-1482569-supplementary/Supplementary Table S1.pdf]

Supplementary Table S1: Characteristics of Included studies.

Study Information				Sample Characteristics						Characteristics of the Scale Used		
**Author (Year)**	**Country**	**Study Design**	**Study Setting**	**Sampling Method**	**Sample Source**	**Population Involved**	**Sample Size**	**Age [Mean/ Median/ Range (sd/iqr)]**	**Female (%)**	**Version**	**Number of Items**	**Mode of Administration**
Abbamonte et al., 2021 [31]	South Africa	Trial	Rural and urban	NR	Community health centres	Pregnant women living with HIV	187	27.91 (5.90)	100.0	Full version	40	ACASI
Anakwa et al., 2021 [32]	Ghana	Cross-sectional	Urban	Non random	HIV clinics in two hospitals	General population of PLWH	235	35.94 (10.26)	51.1	Personalized stigma, disclosure concerns and negative self-image subscales	16	NR
Andrinopoulos et al., 2011 [33]	USA	Cohort	Urban	NR	HIV clinics	Adolescent girls and young women living with HIV	179	21.0 (15–24)	100.0	Two sub-scales	NR	ACASI
Aristegui et al., 2021 [34]	Argentina	Trial	NR	NR	Domiciliary or community-based testing centers	Transgender women living with HV	61	29.87 (6.51)	100.0	Full version	40	NR
Arshi et al., 2020 [35]	Iran	Cross-sectional	Urban	Random	HIV-positive clubs and counselling centres	General population of PLWH	163	37.5 (10.3)	46.0	Full version	40	NR
Ataro et al., 2020 [36]	Ethiopia	Cross-sectional	Urban	Non random	Hospital	General population of PLWH	412	42.60 (9.20)	50.0	Abbreviated version	32	Interviewer-administered

Baik et al., 2020 [37]	USA	Cross-sectional	NR	NR	HIV clinics, community centers and online	General population of PLWH	281	51.00 (10.10)	51.6	Personalized stigma subscale	3	NR
Bennett et al., 2016 [38]	USA	Cross-sectional	Urban	NR	HIV clinic	Youth living with HIV	88	18.3 (3.0)	44.0	Personalized stigma sub-scale	18	Self- and interviewer-administered
Berger et al., 2001 [20]	USA	Development and validation	Urban and rural	Non random	Sites serving PLWH	General population of PLWH	318	37.0 (7.7)	19.4	Full version	40	Self-administered
Bint-E-Saif & Shahzad, 2020 [39]	Pakistan	Validation	Urban	Non-random	HIV/AIDS treatment centre	Injectable drug users living with HIV	150	31.65 (5.88)	NR	Full version	40	NR
Blake et al., 2017 [6]	USA	Cross-sectional	Urban	NR	HIV clinic	General population of PLWH	180	45.4 (11.0)	36.0	Internalized stigma sub-scale	7	Self-administered
Boyes et al., 2013 [40]	South Africa	Validation	Peri-urban	NR	Schools, NGOs, and at home	Adolescents orphaned by AIDS	723	16.9 (2.5)	49.7	Abbreviated version	24	Interviewer-administered
Brener et al., 2013 [41]	Australia	Cross-sectional	NR	Non random	Internet-based sites and community	General population of PLWH	697	43.8 (10.3)	4.6	Abbreviated version	35	Online
Brown et al., 2016 [42]	USA	Cross-sectional	Urban	NR	ASO, hospital, and a University clinical trials unit	Women living with HIV	125	37.8 (9.5)	100.0	Original version	40	NR
Brown et al., 2020 [43]	USA	Cross-sectional	Urban	NR	ASOs, HIV-related venues and online	General population of PLWH	346	18 – >50	44.8	Full version	40	NR
Brown et al., 2020 [44]	USA	Cross-sectional	Urban	NR	Immunology clinic	Older adults living with HIV	156	>50, 84.6% aged 50 – 64 years	34.0	Abbreviated version	12	NR
Bunn et al., 2007 [45]	USA	Validation	NR	Non random	Infectious disease clinics, ASO, and community	General population of PLWH	157	43.2 (9.0)	28.7	Abbreviated version	32	Self-administered

Buseh et al., 2008 [46]	USA	Cross-sectional	Urban	Non random	CBO	Men living with HIV	55	48.8 (7.7)	-	Full version	40	Interviewer-administered
Caliari et al., 2017 [47]	Brazil	Cross-sectional	NR	NR	HIV clinic	General population of PLWH	258	NR; however, 36.0% were between 40–49 years	43.8	Full version	40	Interviewer-administered
Cama et al., 2015 [48]	Australia	Cross-sectional	NR	Non random	HIV organizations and community	General population of PLWH	697	43.8 (10.3)	4.6	Abbreviated version	35	Online
Carrizosa et al., 2010 [49]	Mexico	Cross-sectional	Urban	Non random	HIV clinics	General population of PLWH	342	38.2 (9.9)	32.2	Abbreviated version	6	Interviewer-administered
Cederbaum et al., 2017 [50]	USA	Cross-sectional	Urban	Non random	ASO	Women living with HIV	46	47.4 (9.6)	100.0	Personalized stigma and disclosure concern sub-scales	28	Interviewer-administered
Cernigliaro et al., 2016 [51]	Dominican Republic	Cross-sectional	Urban	Non random	HIV clinics and community	FSW living with HIV	268	36 (18–61)	100.0	Abbreviated version	7	Interviewer-administered
Charles et al., 2012 [52]	India	Cross-sectional	NR	Non random	Community	General population of PLWH	400	NR; however, 55.5% were between 31–40 years	53.0	Full version	40	Interviewer-administered
Chen et al., 2020 [53]	China	Cohort	Urban	NR	CDC center	MSM living with HIV	112	30.00 (8.00)	-	Negative self-image subscale	5	NR
Clum et al., 2009 [54]	USA	Cross-sectional	Urban	NR	HIV care sites	Adolescent girls and young women living with HIV	147	20.6 (2.2)	100.0	Disclosure concerns and negative self-image sub-scales	23	ACASI

Cluver et al., 2008 [55]	South Africa	Cross-sectional	Urban	Non random	Schools and organizations	Adolescents orphaned by AIDS	425	13.7 (2.5)	50.6	Abbreviated version	4	Self- and interviewer-administered
Colbert et al., 2010 [56]	USA	Cross-sectional	NR	NR	NR	General population of PLWH	183	40.7 (7.8)	35.5	Full version	40	NR
Crockett et al., 2020 [57]	USA	Cohort	Urban	NR	HIV clinics	Women living with HIV	1,364	49.00 (9.00)	100.0	Negative self-image subscale	7	Interviewer-administered
Cuca et al., 2017 [58]	USA	Cross-sectional	NR	Non random	HIV clinics and ASO	Women living with HIV	135	48.2 (8.9)	100.0	Full version	40	Interviewer-administered
Deering et al., 2021 [59]	Canada	Cohort	Urban	NR	Referrals from ASOs, HIV care providers, peer navigators and clinical outreaches	Women living with HIV	215	46 (39–53)	100.0	Abbreviated version	9	NR
den Daas et al., 2019 [60]	Netherlands	Cross-sectional	Urban	NR	HIV clinics	General population of PLWH	170	NR: however, participants were >18 years	5.9	Abbreviated version	10	Online
Dowshen et al., 2009 [24]	USA	Cross-sectional	NR	Non random	Community	Young MSM living with HIV	42	21.3	-	Full version	40	NR
Drews et al., 2021 [61]	Germany	Cross-sectional	NR	NR	NR	Older adults living with HIV	839	56.9 (6.3)	11.8	Negative self-image subscale	6	Self-administered
Durteste et al., 2019 [62]	Ukraine	Cross-sectional	Urban	NR	HIV clinics	Youth living with HIV	204	19.0 (15.4–23.0)	52.0	Abbreviated version	NR	Online
Emlet et al., 2007 [63]	USA	Validation	NR	Non random	ASO, public health and infectious disease clinics, and medical centres	Older PLWH	25	56.1 (5.8)	32.0	Full version	40	Interviewer-administered
Emlet et al., 2015 [64]	Canada	Cross-sectional	Urban	NR	HIV study sites	Older PLWH	960	22-86	18.1	Abbreviated version	16	NR

Fair et al., 2010 [65]	USA	Cross-sectional	Urban	NR	ASO	General population of PLWH	38	43.6	44.7	Full version	40	Self-administered
Fekete et al., 2018 [66]	USA	Cross-sectional	NR	Non random	HIV advocacy centres, social services agencies, and community	General population of PLWH	181	42.8 (11.0)	24.9	Negative self-image sub-scale	13	Online
Felker-Kantor et al., 2019 [67]	USA	Cohort	Urban	Non random	NR	General population of PLWH	380	NR; however, 45.3% were between 50–59 years	34.5	Full version	40	NR
Filiatreau et al., 2010 [68]	South Africa	Cross-sectional	Rural	NR	University and research council's research site	Adolescent girls and young women living with HIV	79	20	100.0	Full version	40	Interviewer-administered
Franke et al., 2010 [69]	Peru	Validation	Urban	NR	Health districts	General population of PLWH	130	30.5 (26–37)	53.8	Abbreviated version	21	Interviewer-administered
Fuster-RuizdeApodaca et al., 2015 [70]	Spain	Validation	NR	NR	Health facilities	General population of PLWH	597	43.4	30.5	Abbreviated version	30	Self-administered
Galvan et al., 2008 [71]	USA	Cross-sectional	Urban	Non random	Social service agencies	General population of PLWH	283	16–78	25.8	Full version	40	Interviewer-administered
Gamarel et al., 2020 [72]	USA	Cross-sectional	Urban	Non random	ASO, CBO, primary care clinics and community	Adolescents and young adults living with HIV	109	23.7 (3.7)	19.3	Negative self-image sub-scale	13	Online
Gao et al., 2018 [73]	China	Cross-sectional	Urban	Non random	HIV clinics	General population of PLWH	520	34.5 (11.9)	6.3	Abbreviated version	32	Interviewer administered

Golub et al., 2009 [74]	USA	Cross-sectional	Urban	Non random	HIV/AIDS centre	Sexually active PLWH	38	47.7 (7.5)	36.8	Disclosure concerns sub-scale	4	ACASI
Gonzalez et al., 2009 [76]	USA	Cross-sectional	Urban and rural	Non random	ASO, HIV clinics and community	General population of PLWH	200	43.2 (8.7)	27.5	Abbreviated version	32	Interviewer administered
Gonzalez et al., 2011 [75]	USA	Cross-sectional	Urban and rural	Non random	ASO, HIV clinics and community	Men living with HIV	36	44.3 (8.1)	0.0	Abbreviated version	32	Interviewer administered
Grov et al., 2010 [77]	USA	Cross-sectional	Urban	Non-random	ASO, HIV clinics, and community	Older PLWH	914	54.0 (52–58)	28.9	Full version	40	Self-administered
Ha et al., 2019 [78]	India	Cross-sectional	Urban	Non-random	ART centres	Alcohol consuming men living with HIV	940	41.5	-	Abbreviated version	16	NR
Halkitis et al., 2014 [79]	USA	Cross-sectional	Urban	Non-random	Community	Older MSM living with HIV	199	55.4 (4.6)	-	Abbreviated version	15	NR
Harper et al., 2014 [80]	USA	Trial	Urban	NR	HIV clinics	Adolescents and young adults living with HIV	50	19.2 (2.3)	44.0	Full version	40	ACASI
Harris et al., 2020 [81]	USA	Cross-sectional	NR	NR	ASOs and referrals	African-American older adults living with HIV	35	58.3 (5.4)	25.7	Full version	40	Self-administered
Holzemer et al., 2009 [82]	Kenya, South Africa, Puerto Rico, and USA	Cross-sectional	Urban	Non-random	HIV clinics and HIV-focused community settings	General population of PLWH	726	42.7 (9.5)	45.4	Full version	40	Self-administered
Hosek et al., 2018 [83]	USA	Trial	Urban	Non-random	HIV clinics	Youth living with HIV	103	20.2 (2.0)	19.4	Full version	40	ACASI
Huang et al., 2020 [84]	USA	Cross-sectional	Urban	Non random	Wellness center, Chinese-American planning council and	General population of Asian-Americans PLWH	69	51.0 (10.5)	21.7	Full version	40	ACASI

					HIV/AIDS community health center						
Hubach et al., 2015 [85]	USA	Cross-sectional	Rural	Non-random	CBO	MSM living with HIV	100	42.6 (11.8)	-	Full version	40	Online
Hussen et al., 2015 [86]	USA	Cross-sectional	Urban	NR	HIV clinics	Young MSM living with HIV	132	20.9 (1.9)	-	Negative self-image sub-scale	13	ACASI
Hutson et al., 2018 [87]	USA	Cross-sectional	NR	NR	CBO and HIV clinics	General population of PLWH	216	44.9 (12.4)	25.0	Full version	40	Self-administered or online
Ibrahim et al., 2020 [88]	Indonesia	Cross-sectional	Urban	Non random	HIV clinics	General population of PLWH	122	32.04 (4.93)	58.2	Full version	40	NR
Imaryati et al., 2019 [25]	Indonesia	Cross-sectional	NR	Non-random	HIV clinic	Women living with HIV	120	NR; however, 81.7% were between 18–40 years	100.0	Full version	40	Self-administered
Ivanova et al., 2012 [89]	Canada	Cross-sectional	NR	Non-random	ASO, HIV clinics and community health centres	Women living with HIV	361	37.8 (7.6)	100.0	Concern with public attitudes sub-scale	21	Self-administered
Jaworsky et al., 2018 [90]	Canada	Cross-sectional	Rural	Non-random	ASO, HIV clinics and community health centres	Women living with HIV	701	41.0 (10.4)	100.0	Abbreviated version	10	Online
Jeyaseelan et al., 2013 [91]	India	Validation	Urban and semi-rural	Non-random	HIV networks	General population of PLWH	250	36.8 (5.2) for males 32.8 (6.1) for females	50.4	Full and abbreviated versions	40 and 25	Interviewer-administered
Jimenez et al., 2010 [92]	Puerto Rico	Validation	NR	Non-random	HIV clinics	General population of PLWH	216	45 (8.9) for males 41 (8.2) for females	51.4	Abbreviated version	17	NR

Johnson et al., 2016 [93]	USA	Validation	Urban	NR	Case management agencies	General population of racial minority PLWH	110	46.1 (11.0)	52.7	Abbreviated version	10	NR
Kaai et al., 2010 [94]	Kenya	Trial	Urban	NR	HIV clinics	General population of PLWH	183	37.4 (7.9)	63.0	Abbreviated version	16	Interviewer-administered
Kagiura et al., 2020 [95]	Japan	Validation	NR	NR	Hospitals	General population of PLWH	463	47.7 (10.7)	4.2	Abbreviated version	9	Self-administered
Kamen et al., 2016 [96]	USA	Cross-sectional	Urban	NR	NR	General population of PLWH	334	45.9 (23–67)	26.0	Full version	40	ACASI
Kamitani et al., 2018 [97]	USA	Validation	Urban	Non-random	CBO	General population of PLWH	83	48.2 (4.8) in phase 1 46.8 and 48.7 (2.4, 6.1 in phase 2 44.4 (9.5) in phase 3	9.6	Abbreviated version	13	Self-administered
Kang et al., 2017 [98]	Rwanda	Cross-sectional	Rural	NR	Community	General population of PLWH	14	51.0	71.4	Full version	40	Interviewer-administered
Kerrigan et al., 2017 [100]	Brazil	Cross-sectional	Urban	random	Public health facilities	General population of PLWH	900	41.0	32.5	Negative self-image sub-scale	8	Interviewer-administered
Kerrigan et al., 2021 [99]	Dominican Republic and Tanzania	Cross-sectional	Urban	Non random	Private offices located in universities in respective countries	FSW living with HIV	409	39.11 (9.02) in Dominican Republic 31.69 (7.44) in Tanzania	100.0	Abbreviated version	8	NR
Lacombe-Duncan et al., 2021 [101]	Canada	Cross-sectional	Urban	Non random	HIV clinics, ASOs, CBOs	Transgender women living with HIV	53	41.1 (10.3)	100.0	Personalized stigma, negative self-image and disclosure	7	Interviewer-administered

										concerns subscales		
Li et al., 2014 [103]	Thailand	Cross-sectional	Urban	NR	Community hospital	General population of PLWH	128	44.9 (9.0)	59.4	Abbreviated version	10	Interviewer-administered
Li et al., 2014 [105]	China	Cross-sectional	Rural and urban areas	Non-random	HIV clinics	General population of PLWH	161	46.6 (10.7)	44.7	Full version	40	NR
Li et al., 2016 [102]	USA	Cross-sectional	Urban	NR	NR	MSM living with HIV	297	41.8 (11.1)	-	Abbreviated version	21	ACASI
Li et al., 2016 [104]	China	Cross-sectional	Rural and urban	NR	HIV organization	MSM living with HIV	266	34.2 (9.3)	-	Full version	40	Self-administered
Lin et al., 2010 [106]	China	Cross-sectional	Rural	NR	AIDS orphanages, children's homes and community	Children affected by AIDS and comparison peers	1625	12.9 (2.2)	49.0	Abbreviated version	10	Interviewer-administered
Lindberg et al., 2014 [107]	Sweden	Validation	Urban	NR	Infectious disease clinic of a hospital	General population of PLWH	194	48.8 (11.7)	43.8	Abbreviated version	39	Self-administered
Liu et al., 2014 [109]	China	Cross-sectional	Urban	NR	CDC centre	General population of PLWH	290	32.8 (9.5)	20.3	Full version	40	Interviewer-administered
Liu et al., 2018 [108]	China	Cross-sectional	NR	NR	Hospital	Men living with HIV	220	NR; however, 35% were ≥ 41 years	-	Abbreviated version	20	Interviewer-administered
Logie et al., 2018 [110]	Canada	Cross-sectional	NR	Non-random	Community	Women living with HIV	1424	43 (35–50)	100.0	Abbreviated version	10	Interviewer administered
Luz et al., 2020 [111]	Brazil	Validation	NR	Non random	Online from 3 platforms (Grindr, social media, and Hornet)	General population of PLWH	2,102	38.6 (10.1) for Grindr sample 43.7 (12.3) for social media sample	3.0	Abbreviated version	12	Self-administered

								37.8 (9.9) for Hornet sample				
Lyimo et al., 2014 [112]	Tanzania	Cross-sectional	Rural	NR	HIV clinics	General population of PLWH	158	43.8 (10.3)	69.6	Abbreviated version	5	Interviewer-administered
Magidson et al., 2017 [113]	South Africa	Cross-sectional	Peri-urban	NR	HIV clinics	General population of PLWH	101	35.0 (7.0)	82.2	Full version	40	Self-administered
Mahlomaholo et al., 2021 [114]	Lesotho	Cross-sectional	NR	NR	ART centers within correctional institutions	Inmates living with HIV	402	>18; 51.5% aged ≥ 35 years	11.7	Abbreviated version	12	Interviewer-administered
Mao et al., 2018 [115]	China	Cross-sectional	NR	Random	NR	General population of PLWH	1254	38.9 (8.3)	39.9	Abbreviated version	16	NR
Martiana et al., 2019 [116]	Indonesia	Cross-sectional	NR	Non-random	Hospitals and public health centres	MSM living with HIV	175	29.4 (6.5)	-	Full version	40	NR
Martinez et al., 2012 [117]	USA	Cohort	Urban	NR	HIV clinics	Adolescent girls and young women living with HIV	60	20.6 (2.0)	100.0	Disclosure concerns and negative self-image sub-scales	23	NR
Mason et al., 2010 [118]	USA	Development and validation	Urban	NR	NR	Adolescents affected by HIV	35	15.9 (1.9)	54.3	New scale to measure stigma by association	23	ACASI
Mi et al., 2021 [119]	China	Cross-sectional	Urban and rural	Random	HIV clinics and community health centers	General population of PLWH	1,104	37.05 (5.90)	41.3	Negative self-image and concerns with public attitudes subscales	14	Self or interviewer-administered
Miller et al., 2011 [120]	USA	Cross-sectional	NR	NR	ASO, medical clinics and community	General population of PLWH	203	43.2	27.5	Enacted stigma and disclosure concerns sub-scales	18	ACASI

Montano et al., 2020 [121]	Colombia	Validation	Urban	NR	HIV clinic	General population of PLWH	105	36.6 (9.8)	27.6	Abbreviated version	7	Interviewer-administered
Mukherjee et al., 2017 [122]	India	Cross-sectional	Rural and urban	Non-random	HIV clinics	General population of PLWH	120	NR	53.3	Full version	40	Interviewer-administered
Murphy et al., 2006 [123]	USA	Cohort	NR	NR	ASO and primary care sites	Mothers living with HIV and their uninfected adolescents	236	39.2 (5.8) for mothers 13.0 (1.8) for adolescents	50.0 for adolescents	Abbreviated version	19	Interviewer-administered
Murphy et al., 2018 [124]	UK and Ireland	Cross-sectional	NR	Non-random	ASO, sexual health organizations, and community	MSM living with HIV	-213 in the UK -65 in Ireland	44 (38–51) in the UK 43 (33–49) in Ireland	-	Abbreviated version	16	Online
Mutumba et al., 2017 [125]	Uganda	Cross-sectional	Urban	Non-random	Clinical research centre	Adolescents living with HIV	464	15.6 (2.4)	53.0	Abbreviated version	21	Interviewer-administered
Nabunya et al., 2020 [126]	Uganda	Cross-sectional	Rural	NR	Health centres	Adolescents living with HIV	702	12.42 (1.98)	56.4	Abbreviated version	9	Interviewer-administered
Newman et al., 2012 [127]	DRC	Cross-sectional	Urban	NR	Hospital	HIV-positive caregivers and adult relatives of HIV-positive children	275	35 (30–40)	84.0	Abbreviated version	4	Interviewer-administered
Nobre et al., 2018 [128]	Finland	Cross-sectional	Urban	Non-random	Infectious disease clinic, HIV support groups, and a deaconess institute	General population of PLWH	440	47.5	22.8	Abbreviated version	3	Self-administered
Nyongesa et al., 2019 [131]	Kenya	Cross-sectional	Rural	Non-random	HIV clinic	General population of PLWH	450	42.7 (9.7)	79.1	Abbreviated version	12	Interviewer-administered

Nyongesa et al., 2020 [130]	Kenya	Cross-sectional	Rural	Non-random	HIV clinic	General population of PLWH	450	42.7 (9.7)	79.1	Abbreviated version	12	Interviewer-administered
Nyongesa et al., 2021 [129]	Kenya	Cross-sectional	Rural and urban	Non-random	HIV clinics	Young people living with HIV	812	20.9 (2.1)	50.7	Abbreviated version	12	ACASI
Oke et al., 2019 [132]	Nigeria	Cross-sectional	NR	Random	HIV clinic	General population of PLWH	386	41.2 (9.1)	83.4	Full version	40	Self-administered
Olley et al., 2016 [134]	Nigeria	Cross-sectional	Urban	NR	HIV clinic	General population of PLWH	139	39.6 (10.3)	64.7	Personalized stigma sub-scale	16	Self-administered
Olley et al., 2017 [133]	Nigeria	Cross-sectional	Urban	Non-random	Hospitals	General population of PLWH	502	36.7 (9.4)	62.7	Personalized stigma sub-scale	16	Self-administered
Palar et al., 2018 [135]	USA	Cross-sectional	Urban	NR	Research sites	Women living with HIV	1317	48.7 (8.7)	100.0	Negative self-image sub-scale	7	Interviewer-administered
Patel et al., 2009 [136]	Zimbabwe	Cross-sectional	Urban	NR	Opportunistic infections clinic	Women living with HIV	200	36.4 (8.3)	100.0	Abbreviated version	11	Interviewer-administered
Pearson et al., 2009 [137]	Mozambique	Trial	Urban	NR	HIV clinic	General population of PLWH	277	35.8 (8.9)	56.3	Abbreviated version	21	NR
Peltzer et al., 2018 [138]	South Africa	Trial	Rural	NR	Community health centres	Pregnant women living with HIV	683	28.4 (5.8)	100.0	Full version	40	ACASI
Porter et al., 2017 [139]	USA	Cross-sectional	Urban	NR	Health facilities and community	Older PLWH	914	55.5 (4.9)	29.0	Full version	40	NR
Przybyla et al., 2013 [140]	USA	Trial	NR	NR	NR; authors refer to a previous study for study details	General population of PLWH	341	42.2 (9.0)	34.0	Abbreviated version	7	NR
Qin et al., 2019 [141]	China	Cross-sectional	Rural and urban	Non-random	Medical healthcare organizations	Pregnant women living with HIV	194	25.1 (5.8)	100.0	Full version	40	Interviewer-administered

Quinn et al., 2017 [142]	USA	Trial	NR	NR	Health centre and university hospital	Young MSM living with HIV	92	23.9 (2.9)	-	Abbreviated version	10	Interviewer-administered
Rao et al., 2008 [144]	USA	Validation	NR	Rural and semi-rural	ASO and HIV clinics	General population of PLWH	541	NR	33.0	Full version	40	Self-administered
Rao et al., 2012 [143]	China	Trial	Urban	Urban	HIV clinic and hospital-based HIV support groups	General population of PLWH	120	36.0 (8.0)	18.0	Abbreviated personalized stigma sub-scale	5	Interviewer-administered
Rasoolinajad et al., 2018 [145]	Iran	Cross-sectional	Urban	Non-random	Infectious diseases and behavioural health clinic	General population of PLWH	450	37.3 (8.3)	40.9	Full version	40	NR
Reinius et al., 2017 [147]	Sweden	Development and validation	NR	Non-random	HIV care centres	General population of PLWH	880	47.9	26.0	Abbreviated version	12	Self-administered
Reinius et al., 2018 [146]	Sweden, India, and USA	Validation	Urban and semi-rural	Non-random in India, NR in Sweden, and USA	HIV clinics, CBO, infectious disease clinic, and HIV networks	General population of PLWH	1036	41.0 (11.0)	40.0	Abbreviated version	32	Self- and interviewer-administered
Rendina et al., 2012 [148]	USA	Cross-sectional	Urban	Non-random	Community	MSM living with HIV	127	NR; however, 51.1% were aged between 30–44 years	-	Disclosure concerns and negative self-image sub-scales	15	Self-administered
Rice et al., 2017 [149]	USA	Cross-sectional	Urban	NR	HIV clinic	General population of PLWH	196	44.9 (11.0)	36.7	Negative self-imaged sub-scale	7	Self-administered
Riggs et al., 2007 [150]	USA	Cross-sectional	Urban	Non-random	ASO	General population of PLWH	288	41.5 (8.4)	48.0%	Full version	40	Self-administered

Rongkavilit et al., 2010 [22]	Thailand	Validation and development	Urban	Non-random	HIV clinics	Youth living with HIV	70	22.8 (2.1)	58.6	Full and abbreviated versions	40 and 12	Interviewer- and self-administered
Rubtsova et al., 2021 [151]	USA	Cohort	Urban	NR	NR	Older women living with HIV	356	56.5 (5.2)	100.0	Negative self-image subscale	7	Interviewer-administered
Rueda et al., 2011 [152]	Canada	Cross-sectional	Urban	NR	Clinical research sites	General population of PLWH	825	47.3 (9.9)	16.0	Abbreviated version	16	Self-administered
Schensul et al., 2021 [153]	India	Trial	Urban	NR	ART centres	Men living with HIV	940	42.9 (8.3)	-	Abbreviated version	16	NR
Seb-Akahomen et al., 2019 [154]	Nigeria	Cross-sectional	Urban	NR	HIV clinic	General population of PLWH	410	40.4 (9.8)	75.9	Full version	40	NR
Seghatol-Eslami et al., 2017 [155]	USA	Cross-sectional	Urban	NR	HIV clinic	General population of PLWH	180	45.4 (11.0)	36.0	Negative self-image sub-scale	7	NR
Sereda et al., 2020 [156]	Ukraine	Cross-sectional	Urban	Non-random	Healthcare facilities	General population of PLWH	191	40.0 (7.0)	25.0	Abbreviated version	11	Interviewer-administered
Shamsaei et al., 2020 [157]	Iran	Trial	Urban	NR	HIV clinic	Women living with HIV	55	38.5 (8.6)	100.0	Full version	40	NR
Shokoohi et al., 2019 [158]	Canada	Cross-sectional	Urban	NR	Community	Women living with HIV	1422	24.8 (10.6)	100.0	Abbreviated version	3	Interviewer-administered
Shrestha et al., 2017 [159]	Malaysia	Trial	Urban	NR	HIV unit within a prison	Incarcerated men living with HIV	301	38.9 (6.8)	-	Full version	40	Interviewer-administered
Song et al., 2016 [160]	China	Cross-sectional	Urban	Non-random	Infectious disease clinic	MSM living with HIV	125	30.7 (19–67)	-	Abbreviated version	20	Interviewer-administered
Storholm et al., 2013 [161]	USA	cross-sectional	Urban	NR	ASO, HIV clinics, and community	Older PLWH	904	55.5 (4.9)	29.2	Full version		Self-administered
Sumari-de Boer et al., 2013 [162]	Netherlands	Cross-sectional	Urban	NR	HIV clinic	Immigrant and indigenous PLWH	202	43.3 (10.9)	46.0	Personalized stigma and disclosure	18	Interviewer-administered

									concern sub-scales		
Tanney et al., 2012 [163]	USA	Trial	Urban	NR	Adolescent medical clinics	Youth living with HIV	186	NR	NR	Abbreviated version	10	CAPI
Thomas et al., 2005 [164]	India	Cross-sectional	Rural and urban	NR	NGO, ANC clinics, STD clinics	General population of PLWH	203	NR; however, 68% were aged between 26–39 years	50.2	Full version	40	NR
Tomassilli et al., 2013 [165]	USA	Cross-sectional	Urban	NR	NR; authors refer to a previous study for study information	General population of PLWH	60	47.8	33.3	Abbreviated version	15	ACASI
Toth et al., 2016 [166]	USA	Cross-sectional	Urban	NR	Dental clinics	General population of PLWH	100	25-64	27.0	Full version	40	NR
Turan et al., 2016 [169]	USA	Cohort	Urban	NR	HIV clinics, rehabilitation programs, women support groups, and community	Women living with HIV	1168	49.1 (8.6)	100.0	Negative self-image sub-scale	7	Interviewer-administered
Turan et al., 2017 [167]	USA	Cross-sectional	Urban	NR	HIV clinic	General population of PLWH	203	44.8 (11.1)	36.5	Concern with public attitudes and negative self-image sub-scales	13	Self-administered
Turan et al., 2017 [168]	USA	Cross-sectional	urban	NR	HIV clinics, rehabilitation programs, women support groups, and community	Women living with HIV	1356	48.8 (9.1)	100.0	Negative self-image sub-scale	7	Interviewer-administered

Valenzuela et al., 2015 [170]	Peru	Case-control	Urban	Non-random	HIV clinic	General population of PLWH	176	NR; however, 39.2% were aged between 26–35 years	39.8	Abbreviated version	20	Interviewer-administered
Valle et al., 2015 [171]	Mexico	Validation	NR	Non-random	NGO, CBO	MSM living with HIV	75	35.0 (7.4)	-	Abbreviated version	21	Online
Van der Kooji et al., 2021 [172]	Netherlands	Cross-sectional	Urban	NR	Hospital	General population of PLWH	1,704	50.7 (10.9)	6.0	Abbreviated version	10	Interviewer-administered
Varni et al., 2012 [173]	USA	Cross-sectional	Rural	NR	ASO, medical centres, and community	General population of PLWH	193	43.2 (8.7)	27.5	Abbreviated version	32	NR
Voisin et al., 2017 [174]	USA	Trial	NR	NR	University hospital and health centre	Young MSM living with HIV	92	23.8 (2.9)	-	Abbreviated version	4	ACASI and CAPI
Wang et al., 2019 [175]	China	Cross-sectional	Rural and urban	NR	Medical centre	General population of PLWH	520	34.5 (12.0)	6.3	Abbreviated version	24	Self-administered
Wiklander et al., 2013 [23]	Sweden	Validation	NR	NR	HIV clinics and community	Children living with HIV	58	13.9 (2.5)	46.6	Abbreviated version	8	Self-administered
Williams et al., 2020 [176]	USA	Cross-sectional	NR	NR	NR; authors refer to a previous study for study information	General population of PLWH	603	49.0 (0.6)	30.0	Abbreviated version	10	Interviewer-administered
Wolitski et al., 2009 [177]	USA	Trial	Urban	Non-random	Community	Homeless and unstably housed PLWH	637	NR; however, 48.2% were aged between 40–49 years	30.3	Abbreviated version	6	Interviewer-administered
Wright et al., 2007 [178]	USA	Validation	Urban	NR	HIV clinic	Youth living with HIV	48	16-25	46.0	Abbreviated version	10	Self-administered
Wu et al., 2008 [179]	Peru	Cross-sectional	Urban	Non-random	Hospitals	Impoverished women	78	30.7 (6.9)	100.0	Full version	40	Interviewer-administered

						living with HIV						
Xiao et al., 2015 [180]	China	Cross-sectional	Rural and urban	random	Community	General population of PLWH	125	NR; however, 52.8% were >37 years	30.4	Abbreviated version	14	Self- and interviewer-administered
Yang et al., 2019 [181]	China	Cross-sectional	Rural and urban	Non-random	NR	General population of PLWH	318	37 (33–45)	38.7	Full version	40	Self-administered
Yang et al., 2020 [182]	China	Cohort	NR	NR	HIV clinics	MSM living with HIV	193	30.1 (7.0)	-	Negative self-image sub-scale	12	Interviewer-administered
Yigit et al., 2020 [183]	USA	Cohort	Urban	NR	HIV clinics	General population of PLWH	186	36.33 (12.78)	19.4	Negative self-image subscale	7	Self-administered
Yu et al., 2019 [184]	Taiwan	Development and validation	NR	Non-random	HIV clinics	General population of PLWH	540	29.8 (7.7)	0.6	Abbreviated version	18	NR
Zeligman et al., 2016 [185]	USA	Cross-sectional	NR	NR	ASO and support groups	General population of PLWH	126	49.0 (10.8)	31.0	Full version	40	Self-administered
Zeng et al., 2018 [186]	China	Cross-sectional	Urban	Non-random	HIV clinic	General population of PLWH	411	39.0 (9.1)	31.4	Abbreviated version	14	Self-administered
Zhang et al., 2015 [188]	China	Cross-sectional	Rural and urban	Random	Local CDC and community health centres	General population of PLWH	2987	42.5 (12.8)	37.2	Negative self-image sub-scale	8	Self- and interviewer-administered
Zhang et al., 2016 [187]	China	Cross-sectional	Rural and urban	Random	Local CDC and community health centres	General population of PLWH	2987	42.9 (12.8)	37.0	Abbreviated version	16	Self- and interviewer-administered
Zhou et al., 2017 [189]	China	Cross-sectional	Rural and urban	Random	Local CDC and community health centres	General population of PLWH	2095	42.4 (12.4)	38.6	Abbreviated version	14	Self- and interviewer-administered

Zulliger et al., 2015 [190]	Dominican Republic	Cross-sectional	Urban	Non-random	HIV clinics and community	FSW living with HIV	268	36 (30–42)	100.0	Abbreviated version	8	Interviewer-administered

ASO-AIDS service organizations; CBO-Community-based organizations, CDC-Centre for disease control and prevention; FSW- Female sex workers; MSM-Men who have sex with men; NGO-Non-governmental organization; HIV-Human immunodeficiency virus; AIDS-Acquired Immune Deficiency Syndrome; USA-United States of America; UK-United Kingdom; PLWH-People living with HIV; IQR-Interquartile range; SD-Standard deviation; NR-Not Reported.
