# Supplementary material for: Psychometric Properties of the Berger HIV Stigma Scale: A Systematic Review
Source: Int J Environ Res Public Health. 2021 Dec 11;18(24):13074. doi: 10.3390/ijerph182413074 (PMC8701211; doi:10.3390/ijerph182413074)
Supplement: Supplementary file 1 [file ijerph-18-13074-s001.zip › ijerph-1482569-supplementary/Supplementary Table S2.pdf]

Supplementary Table S2: Psychometric properties of the HSS as reported by the observational or experimental studies.

First Author (Year)	Scale Used	Reliability		Validity				
		**Internal Consistency (Cronbach's Alpha Unless Otherwise Stated)**	**Test-Retest Reliability (icc)**	**Construct Validity**	**Convergent Validity**	**Content Validity**	**Face Validity**	**Cross-Cultural Adaptation and/or Validity**
Abbamonte et al., 2021 [31]	40-item scale	0.96 for the overall scale	NR	NR	NR	NR	NR	NR
Anakwa et al., 2021 [32]	16-item (Personalized stigma (5 items), disclosure concerns (6 items) and negative self-image subscales (5 items))	0.71, 0.73, and 0.72 for the subscales, respectively.	NR	NR	NR	NR	NR	NR
Andrinopoulos et al., 2011 [33]	Two subscales	0.89 for the overall scale	NR	NR	NR	NR	NR	NR
Aristegui et al., 2021 [34]	40-item scale	0.95 (omega)	NR	NR	NR	NR	NR	NR
Arshi et al., 2020 [35]	40-item scale	0.83 for the overall scale.	NR	NR	NR	14 experts judged the scale to be valid (content validity of 0.82)	-Face validity index of 0.56	Used previously translated measures.
Ataro et al., 2020 [36]	32-item scale	0.876 for the overall scale. 0.864 – 0.883 for the subscales.	NR	NR	NR	NR	NR	Scale translated to Amharic and Afan Oromo and back-translated to English, then pilot-tested before use.

Baik et al., 2020 [37]	3-item (personalized stigma subscale)	0.85	NR	NR	NR	NR	NR	NR
Bennet et al., 2016 [38]	18-item (personalized stigma sub-scale)	0.93	NR	NR	NR	NR	NR	NR
Blake et al., 2017 [6]	7-item (internalized stigma sub-scale)	0.85	NR	NR	NR	NR	NR	NR
Brener et al., 2013 [41]	35-item scale	0.88 for the overall scale.	NR	NR	NR	NR	NR	NR
Brown et al., 2016 [42]	40-item scale	0.96 for the overall scale.<br>0.91 –0.95 for the subscales.	NR	NR	NR	NR	NR	NR
Brown et al., 2020 [43]	40-item scale	0.95 for the overall sample.<br>0.94 – 0.96 across samples	NR	NR	NR	NR	NR	NR
Brown et al., 2020 [44]	12-item scale	0.87	NR	NR	NR	NR	NR	NR
Buseh et al., 2008 [46]	40-item scale	0.93 for the overall scale	NR	NR	NR	NR	NR	NR
Caliari et al., 2017 [47]	40-item scale	0.95 for the overall scale.<br>0.84 – 0.96 for the subscales	NR	NR	NR	NR	NR	NR
Cama et al., 2015 [48]	35-item scale	0.92 for the overall scale	NR	NR	NR	NR	NR	NR
Carrizosa et al., 2010 [49]	6-item scale	0.70	NR	NR	NR	NR	NR	NR
Cederbaum et al., 2017 [50]	28-item (personalized stigma and	0.96 for personalised stigma sub-scale.	NR	NR	NR	NR	NR	NR

	disclosure concerns sub-scales)	0.87 for disclosure concerns subscale.						
Cernigliaro et al., 2016 [51]	7-item scale	-0.88	NR	NR	NR	NR	NR	NR
Charles et al., 2012 [52]	40-item scale	0.79 for the overall scale.<br>0.62 – 0.85 for the sub-scales.	0.89	NR	NR	NR	NR	NR
Chen et al., 2020 [53]	5-item (negative self-image subscale)	0.92 and 0.90 at baseline and follow-up, respectively	NR	NR	NR	NR	NR	NR
Clum et al., 2009 [54]	23-item (disclosure concerns and negative self-image sub-scales)	0.90 for the overall scale.	NR	NR	NR	NR	NR	NR
Cluver et al., 2008 [55]	4-item scale	0.83	NR	NR	NR	NR	NR	NR
Colbert et al., 2010 [56]	40-item scale	0.95 for the overall scale	NR	NR	NR	NR	NR	NR
Crockett et al., 2020 [57]	7-item (negative self-image subscale)	0.87 at baseline	NR	NR	NR	NR	NR	NR
Cuca et al., 2017 [58]	40-item scale	0.96 for the overall scale	NR	NR	NR	NR	NR	NR
Deering et al., 2021 [59]	9-item scale	0.84 for the overall scale<br>0.79 – 0.88 for the subscales	NR	NR	NR	NR	NR	NR
den Daas et al., 2019 [60]	10-item scale	0.83 for the overall scale	NR	The 4-factor structure of the original scale was replicated with items loading on the relevant sub-scales.	Expected correlation (r) between the scale and depression (0.16), social support (-0.35), sexuality problems (0.33), and	NR	NR	NR

				Sub-scales are intercorrelated, similar to the original scale.	perceived side effects (0.21) P < 0.001 for all correlations			
Dowshen et al., 2009 [24]	40-item scale	0.94 for the overall scale. 0.79 – 0.93 for the sub-scales.	NR	NR	NR	NR	NR	NR
Drews et al., 2021 [61]	6-item (negative self-image subscale	0.89	NR	NR	NR	NR	NR	Used a previously translated version.
Durteste et al., 2019 [62]	10-item scale	0.78	NR	NR	NR	NR	NR	NR
Emlet et al., 2015 [64]	16-item scale	0.90 for the overall scale. 0.82 –0.87 for the subscales.	NR	NR	NR	NR	NR	NR
Fair et al., 2010 [65]	40-item scale	0.89 for the overall scale. 0.77 –0.80 for the sub-scales.	NR	NR	NR	NR	NR	NR
Fekete et al., 2018 [66]	13-item (negative self-image sub-scale)	0.90	NR	NR	NR	NR	NR	NR
Felker-Kantor et al., 2019 [67]	40-item scale	0.95 for the overall scale. 0.84 – 0.94 for the sub-scales.	NR	NR	NR	NR	NR	NR
Filiatreau et al., 2010 [68]	40-item scale	0.90 for the overall scale. 0.59 – 0.90 for the sub-scales.	NR	NR	NR	NR	NR	NR
Galvan et al., 2008 [71]	40-item scale	0.94 for the overall scale.	NR	The 4-factor structure of the original scale was replicated on factor analysis.	NR	NR	NR	NR

		0.82 – 0.93 for the sub-scales.						
Gamarel et al., 2020 [72]	13-item (negative self-image sub-scale)	0.76	NR	NR	NR	NR	NR	NR
Gao et al., 2018 [73]	32-item scale	0.96 for the overall scale	NR	NR	NR	NR	NR	NR
Golub et al., 2009 [74]	4-item (disclosure concerns sub-scale)	0.85	NR	NR	NR	NR	NR	NR
Gonzalez et al., 2009 [76]	32-item scale	0.88 – 0.95 for the subscales.	NR	NR	NR	NR	NR	NR
Gonzalez et al., 2011 [75]	32-item scale	0.95 for the overall scale.<br>0.89 – 0.95 for the subscales.	NR	NR	NR	NR	NR	NR
Grov et al., 2010 [77]	40-item scale	0.96 for the overall scale.	NR	NR	NR	NR	NR	NR
Ha et al., 2019 [78]	16-item scale	0.88	NR	NR	NR	NR	NR	NR
Halkitis et al., 2014 [79]	15-item scale	0.92	NR	NR	NR	NR	NR	NR
Harper et al., 2014 [80]	40-item scale	0.81 –0.93 for the sub-scales.						
Harris et al., 2020 [81]	40-item scale	0.96 for the overall scale<br>0.86 –0.95 for the subscales	NR	NR	NR	NR	NR	NR
Holzemer et al., 2009 [82]	40-item scale	0.96 for the overall scale.	NR	NR	NR	NR	NR	A Spanish version of the scale was developed by forward translation to Spanish and back translation to English.
Hosek et al., 2018 [83]	40-item scale	0.94 for the overall scale.	NR	NR	NR	NR	NR	NR

Huang et al., 2020 [84]	40-item scale	0.95 for the overall scale	NR	NR	NR	NR	NR	NR
Hubach et al., 2015 [85]	40-item scale	0.94 for the overall scale.	NR	NR	NR	NR	NR	NR
Hussen et al., 2015 [86]	13-item (negative self-image sub-scale)	0.90	NR	NR	NR	NR	NR	NR
Hutson et al., 2018 [87]	40-item scale	0.96 for the overall scale.<br>0.89–0.96 for the subscales.	NR	NR	NR	NR	NR	NR
Ibrahim et al., 2020 [88]	40-item scale	0.90 – 0.93	0.89 – 0.92	NR	NR	NR	NR	Used a previously translated and validated version.
Imaryati et al., 2019 [25]	40-item scale	0.94 for the overall scale.	NR	NR	NR	NR	NR	NR
Ivanova et al., 2012 [89]	20-item (concern with public attitudes sub-scale)	0.94	NR	NR	NR	NR	NR	NR
Jaworsky et al., 2018 [90]	10-item scale	0.87 for the overall scale.<br>0.77 – 0.90 for the sub-scales.	NR	NR	NR	NR	NR	NR
Kaai et al., 2010 [94]	16-item scale	0.81 for the overall scale.	NR	NR	NR	NR	NR	The scale was translated to Swahili and back-translated to English; then pretested for accuracy and comprehension before use.
Kamen et al., 2016 [96]	40-item scale	0.95 for the overall scale.	NR	NR	NR	NR	NR	NR
Kang et al., 2017 [98]	40-item scale	0.96 for the overall scale.	NR	NR	NR	NR	NR	The scale was forward translated to Kinyarwanda then back-

								translated to English by a different independent translator.
Kerrigan et al., 2017 [100]	16-item (enacted stigma and negative self-image sub-scales.	0.76 for the enacted stigma sub-scale.<br>0.75 for the negative self-image sub-scale.	NR	NR	NR	NR	NR	NR
Kerrigan et al., 2021 [99]	8-item scale	0.91 in the Dominican Republic<br>0.88 in Tanzania	NR	NR	NR	NR	NR	NR
Lacombe-Duncan et al., 2021 [101]	7-item (personalized stigma (3 items), negative self-image (3 items) and disclosure concerns (2 items) subscales)	0.85 for personalized stigma subscale<br>0.76 for disclosure concerns subscale<br>0.88 for the negative self-image subscale	NR	NR	NR	NR	NR	NR
Li et al., 2014 [103]	10-item scale	0.72 –0.84 for the sub-scales.	NR	NR	NR	NR	NR	The scale was translated to Thai the back-translated to English.
Li et al., 2014 [105]	40-item scale	0.94 for the overall scale.	NR	NR	NR	NR	NR	The scale was revised and translated to Chinese.
Li et al., 2016 [102]	21-item scale	0.96 for the overall scale.	NR	NR	NR	NR	NR	NR
Li et al., 2016 [104]	40-item scale	0.95 for the overall scale.<br>0.78 – 0.93 for the sub-scales.	NR	NR	NR	Content validity index of 0.87.	NR	The scale was translated to Mandarin Chinese then back-translated to English by two bilingual speakers.

Lin et al., 2010 [106]	20-item (perceived public stigma and personalised stigma sub-scales.	0.86 for the perceived stigma sub-scale.<br>0.87 for the personalised stigma sub-scale.	NR	NR	NR	NR	NR	NR
Liu et al., 2014 [109]	40-item scale	0.91 for the overall scale.	NR	NR	NR	NR	NR	NR
Liu et al., 2018 [108]	20-item scale	0.90 for the overall scale.	NR	NR	NR	NR	NR	NR
Logie et al., 2018 [110]	10-item scale	0.85 for the overall scale.	NR	NR	NR	NR	NR	NR
Lyimo et al., 2014 [112]	5-item (negative self-image sub-scale)	0.78	NR	NR	NR	NR	NR	NR
Magidson et al., 2017 [113]	40-item scale	0.93 for the overall scale	NR	NR	NR	NR	NR	NR
Mahlomaholo et al., 2021 [114]	12-item scale	0.78	NR	NR	NR	NR	NR	NR
Mao et al., 2018 [115]	16-item scale	0.90 – 0.91 for the sub-scales.	NR	NR	NR	NR	NR	NR
Martiana et al., 2019 [116]	40-item scale	0.94 for the overall scale.	NR	NR	NR	NR	NR	Used an Indonesian version of the scale.
Martinez et al., 2012 [117]	23-item (disclosure and negative self-image sub-scales.	0.90 for the overall scale.	NR	NR	NR	NR	NR	NR
Mi et al., 2021 [119]	14-item (Negative self-image (9 items) and concerns with public attitudes (5 items) subscales)	0.93	NR	NR	NR	NR	NR	NR
Miller et al., 2011 [120]	18-item (enacted stigma and disclosure concerns	0.82 – 0.95 for the sub-scales.	NR	NR	NR	NR	NR	NR

	sub-scales of the Bunn et al. scale)							
Mukherjee et al., 2017 [122]	40-item scale	0.95 for the overall scale.	NR	NR	NR	NR	NR	An Indian-translated version of the scale was pilot-tested for its reliability and suitability to the cultural setting of the study
Murphy et al., 2006 [123]	19-item scale	0.87 for the overall scale.	NR	NR	NR	NR	NR	NR
Murphy et al., 2018 [124]	16-item scale	0.79 – 0.89 for the sub-scales.	NR	NR	NR	NR	NR	NR
Mutumba et al., 2017 [125]	21-item scale	0.78 for the overall scale.	NR	NR	NR	NR	NR	The scale was blindly translated to Luganda by two independent translators. Members of the study team then reviewed the translations to ensure they captured the conceptual meanings of items, were understandable, and resolved discrepancies. The final choice of the most appropriate translation was based on consensus and consultation with paediatric care providers and ALWH.
Nabunya et al., 2020 [126]	9-item scale	0.74	NR	NR	NR	NR	NR	Scale translated to Luganda and back-translated to English

Newman et al., 2012 [127]	4-item scale	0.63	NR	NR	NR	NR	NR	The scale was translated to French and Lingala, then back-translated to English. Both versions were compared to ensure that all meaning and nuances had been captured.
Nobre et al., 2018 [128]	3-item scale	0.86	NR	NR	NR	NR	NR	NR
Nyongesa et al., 2019 [131]	12-item scale	0.81	NR	NR	NR	NR	NR	NR
Nyongesa et al., 2020 [130]	12-item scale	0.81	NR	NR	NR	NR	NR	Scale was translated to Swahili and back-translated to English by independent translators, then harmonized.
Nyongesa et al., 2021 [129]	12-item scale	0.81	NR	NR	NR	NR	NR	NR
Oke et al., 2019 [132]	40-item scale	0.90 for the overall scale	NR	NR	NR	NR	NR	The scale was translated to Yoruba by two independent translators. A unified version was back-translated to English by two other independent translators, pilot tested, and disagreements resolved.
Olley et al., 2016 [134]	16-item (Personalized stigma sub-scale)	0.54	NR	NR	NR	NR	NR	NR
Olley et al., 2017 [133]	16-item (personalized stigma sub-scale)	0.83	NR	NR	Expected correlation (r) between the scale and measures of	NR	NR	NR

					depression (0.27, P < 0.01) and self-esteem (-0.37, P < 0.01)			
Palar et al., 2018 [135]	7-item (negative self-image sub-scale.	0.90	NR	NR	NR	NR	NR	NR
Patel et al., 2009 [136]	11-item scale	0.80 and 0.74 for the perceived and experienced stigma, respectively.	NR	NR	NR	NR	NR	The scale was translated to Shona and back-translated to English.
Pearson et al., 2009 [137]	21-tem scale	0.87 for the overall scale.<br>0.79 – 0.90 for the sub-scales.	NR	NR	NR	Items were selected from a published and validated scale to ensure content validity.	NR	All items were translated into Portuguese, pretested, and psychometric properties were assessed for cultural appropriateness in Mozambique.
Peltzer et al., 2018 [138]	40-item scale	0.96 for the overall scale.<br>0.79 –0.94 for the sub-scales.		NR	NR	NR	NR	NR
Porter et al., 2017 [139]	40-item scale	0.83 – 0.95 for the sub-scales.	NR	NR	NR	NR	NR	NR
Przybyla et al., 2013 [140]	7-iten (disclosure concerns sub-scale)	0.87	NR	NR	NR	NR	NR	NR
Qin et al., 2019 [141]	40-item scale	0.95 for the overall scale.	NR	NR	NR	NR	NR	NR
Quinn et al., 2017 [142]	10-item scale	0.72 for the overall scale.	NR	NR	NR	NR	NR	NR
Rao et al., 2012 [143]	5-item (personalized stigma sub-scale)	0.84	NR	PCA revealed a one-factor structure that explained 61.4% of the variance.	NR	NR	NR	NR

Rasoolinajad et al., 2018 [145]	40-item scale	0.81 for the overall scale.	NR	NR	NR	NR	NR	NR
Rendina et al., 2012 [148]	15-item (disclosure concerns and negative self-image sub-scales)	0.83 and 0.82 for the two sub-scales, respectively.	NR	NR	NR	NR	NR	NR
Rice et al., 2017 [149]	7-item (negative self-image sub-scale)	0.85	NR	NR	NR	NR	NR	NR
Riggs et al., 2007 [150]	40-item scale	0.83 –0.95 for the sub-scales.	NR	NR	NR	NR	NR	NR
Rubtsova et al., 2021 [151]	7-item (negative self-image subscale	0.90	NR	NR	NR	NR	NR	NR
Rueda et al., 2011 [152]	16-item scale	0.88 for the overall scale.<br>0.78 – 0.88 for the sub-scales.	NR	NR	NR	NR	NR	NR
Schensul et al., 2021 [153]	16-item scale	0.88	NR	NR	NR	NR	NR	NR
Seb-Akahomen et al., 2019 [154]	40-item scale	0.91 for the overall scale.	NR	NR	NR	NR	NR	NR
Seghatol-Eslami et al., 2017 [155]	7-item (negative self-image sub-scale.	0.85	NR	NR	NR	NR	NR	NR
Sereda et al., 2020 [156]	11-item scale	0.75 for the overall scale.	NR	CFA confirmed a 4-factor structure similar to the original scale with a good model fit (CFI=0.969; TLI=0.955; SRMR=0.089	NR	NR	NR	A Ukrainian version of the scale was developed and piloted before being used in the study.
Shamsaei et al., 2020 [157]	40-item scale	0.94 for the overall scale.	NR	NR	NR	NR	NR	NR
Shokoohi et al., 2019 [158]	3-item scale	0.85	NR	NR	NR	NR	NR	NR

Shrestha et al., 2017 [159]	40-item scale	0.90 for the overall scale.						The scale was translated to Bahasa Malaysia then back-translated to English. This version was then adapted to the Malaysian context.
Song et al., 2016 [160]	20-item scale	0.83 for the overall scale.	NR	NR	NR	NR	NR	Used a previously validated Chinese version of the scale.
Storholm et al., 2013 [161]	40-item scale	0.87 for the overall scale.	NR	NR	NR	NR	NR	NR
Sumari-de Boer et al., 2013 [162]	28-item (personalized stigma and disclosure concerns sub-scales)	0.94 and 0.84 for the personalized and disclosure concerns sub-scales, respectively.	NR	NR	NR	NR	NR	The scale was translated to Dutch by two bilingual researchers.<br>Discussions were held to resolve discrepancies.<br>-Items in the final version were clarified through interviews.
Tanney et al., 2012 [163]	10-item scale	0.80 for the overall scale.	NR	NR	NR	NR	NR	NR
Thomas et al., 2005 [164]	40-item scale	0.95 for the overall scale.	NR	NR	NR	NR	NR	Scale was translated to Tamil.
Tomassilli et al., 2013 [165]	15-item scale	0.91 for the overall scale.	NR	NR	NR	NR	NR	NR
Toth et al., 2016 [166]	40-item scale	NR	0.92	NR	NR	NR	NR	NR
Turan et al., 2016 [169]	7-item (negative self-image sub-scale)	0.91	NR	NR	NR	NR	NR	NR
Turan et al., 2017 [167]	13-item (concern with public attitudes and	0.84 and 0.85 for concern with public attitudes and the negative	NR	NR	NR	NR	NR	NR

	negative self-image sub-scales)	self-image sub-scales, respectively.						
Turan et al., 2017 [168]	7-item (negative self-image sub-scale)	0.88	NR	NR	NR	NR	NR	NR
Valenzuela et al., 2015 [170]	20-item scale	0.64 – 0.82 for the sub-scales.	NR	NR	NR	NR	NR	Used a modified, Spanish-language version of the scale.
Van der Kooji et al., 2021 [172]	10-item scale	0.66 – 0.89 for subscales	NR	NR	NR	NR	NR	NR
Varni et al., 2012 [173]	32-item scale	0.90 – 0.97 for the sub-scales.	NR	NR	NR	NR	NR	NR
Voisin et al., 2017 [174]	6-item (personalised stigma and negative self-image sub-scales)	0.72 for the overall scale.	NR	NR	NR	NR	NR	NR
Wang et al., 2019 [175]	24-item scale	0.96 for the overall scale.<br>0.91 – 0.94 for the sub-scales.	NR	NR	NR	NR	NR	Used a previously translated Chinese version of the scale.
Williams et al., 2020 [176]	10-item scale	0.64 – 0.81 for the subscales.	NR	EFA revealed a 3-factor structure with factor loadings ≥40, with no cross-loadings. The first two factors were consistent with two factors in Wright's abbreviated 10-item scale that had a 4-factor structure	NR	NR	NR	NR
Wolitski et al., 2009 [177]	12-item (internal and perceived external stigma sub-scales)	0.92 for the overall scale.<br>0.88 and 0.91 for the subscales, respectively.	NR	EFA confirmed the 2-factor structure with factor loadings above 0.64 in the 2 sub-scales.	NR	NR	NR	NR

Wu et al., 2008 [179]	40-item scale	>80 for the overall scale	NR	NR	NR	NR	NR	NR
Xiao et al., 2015 [180]	14-item scale	0.92 and 0.90 for the sub-scales.	NR	EFA of the 14-items produced two factors (internalised stigma and perceived stigma)	NR	NR	NR	NR
Yang et al., 2019 [181]	40-item scale	0.95 for the overall scale.<br>0.78 –0.93 for the sub-scales.	NR	NR	NR	NR	NR	NR
Yang et al., 2020 [182]	12-item (negative self-image sub-scale)	0.88	NR	NR	NR	NR	NR	NR
Yigit et al., 2020 [183]	7-item (negative self-image subscale	0.77 at baseline and 0.82 at follow-up	NR	NR	NR	NR	NR	NR
Zeligman et al., 2016 [185]	40-item scale	0.96 for the overall scale.	NR	NR	NR	NR	NR	NR
Zeng et al., 2018 [186]	14-item scale	0.93	NR	NR	NR	NR	NR	NR
Zhang et al., 2015 [188]	8-item (negative self-image sub-scale)	0.92	NR	NR	NR	NR	NR	NR
Zhang et al., 2016 [187]	16-item scale	0.93 for the overall scale.<br>0.63 – 0.92 for the overall scale.	NR	NR	NR	NR	NR	NR
Zhou et al., 2017 [189]	14-item scale	0.79 –0.93	NR	NR	NR	NR	NR	NR
Zulliger et al., 2015 [190]	8-item scale	0.87	NR	PCA suggested a 1-factor structure for the 8 items.	NR	NR	NR	NR

PCA-Principal component analysis, CFA-Confirmatory factor analysis; EFA-Exploratory factor analysis; CFI-Comparative fit index; χ2Chi-square; df-Degree of freedom; RMSEA-Root mean square of approximation; SRMR-Standardized root mean square residual; TLI-Tucker-Lewis Index; a- Cronbach alpha; ICC-Intraclass correlation; NR-Not Reported.
