# Supplementary material for: Psychometric Properties of the Berger HIV Stigma Scale: A Systematic Review
Source: Int J Environ Res Public Health. 2021 Dec 11;18(24):13074. doi: 10.3390/ijerph182413074 (PMC8701211; doi:10.3390/ijerph182413074)
Supplement: Supplementary file 1 [file ijerph-18-13074-s001.zip › ijerph-1482569-supplementary/Supplementary Table S3.pdf]

Supplementary Table S3: Quality ratings of the included development and/or validation studies.

First Author	PROM Development	Internal Consistency	Test-Retest Reliability	Construct Validity	Convergent Validity	Divergent Validity	Discriminant Validity	Content Validity	Face Validity	Cross-Cultural Validity
Berger et al., 2001 [20]	Doubtful	Very good	Very good	Adequate	Very good	NR	NR	Adequate	NA	NR
Bint-E-Saif & Shahzad, 2020 [39]	NA	Very good	Adequate	NR	Inadequate	NR	NR	NR	NR	NR
Boyes et al., 2010 [40]	NA	Very good	NR	Adequate	Adequate	NR	Very good	Doubtful	NR	NR
Bunn et al., 2007 [45]	NA	Very good	NR	Adequate	Very good	NR	NR	NR	NR	NR
Emlet et al., 2007 [63]	NA	Very good	NR	NR	Inadequate	NR	NR	Doubtful	NR	NR
Franke et al., 2010 [69]	NA	Very good	NR	Adequate	Very good	NR	NR	NR	NR	NR
Fuster-RuizdeApodaca et al., 2015 [70]	NA	Very good	NR	Very good	Very good	NR	Adequate	Adequate	Doubtful	NR
Jeyaseelan et al., 2013 [91]	NA	Very good	Adequate	Adequate	Very good	NR	NR	Doubtful	Adequate	NR
Jimenez et al., 2010 [92]	NA	Very good	Very good.	Very good	Very good	NR	NR	NR	NR	NR
Johnson et al., 2016 [93]	NA	Very good	NR	Very good	Doubtful	NR	NR	NR	NR	NR
Kagiura et al., 2020 [95]	NA	Very good	NR	Very good	Adequate	NR	NR	NR	NR	NR

Kamitani et al., 2018 [97]	NA	Very good	NR	Adequate	Very good	211	NR	Doubtful	NR	NR
Lindberg et al., 2014 [107]	NA	Very good	NR	Adequate	Inadequate	NR	NR	Doubtful	NR	NR
Luz et al., 2020 [111]	NA	Very good	NR	Very good	NR	NR	Very good	Doubtful	NR	Adequate
Mason et al., 2010 [118]	Doubtful	Very good	NR	NR	NR	NR	NR	Doubtful	NR	NR
Montano et al., 2020 [121]	NA	Very good	Adequate	Very good	Very good	NR	NR	NR	NR	NR
Rao et al., 2008 [144]	NA	NR	NR	NR	NR	NR	NR	NR	NR	Very good
Reinius et al., 2017 [147]	NA	Very good	NR	Adequate	NR	NR	NR	Doubtful	NR	NR
Renius et al., 2018 [146]	NA	NR	NR	NR	NR	NR	NR	NR	NR	Very good
Rongkavilit et al., 2010 [22]	NA	Very good	NR	Adequate	NA	NR	NR	NR	NR	NR
Valle et al., 2015 [171]	NA	Very good	NR	Adequate	Doubtful	NR	Adequate	NR	NR	NR
Wiklander et al., 2013 [23]	NA	Very good	NR	Adequate	Inadequate	NR	NR	NR	NR	NR
Wright et al., 2007 [178]	NA	Very good	NR	Adequate	Very good	NR	NR	NR	NR	NR
Yu et al., 2019 [184]	NA	Very good	NR	Very good	Very good	NR	NR	Adequate	NR	NR

NA – Not Applicable; NR – Not Reported; PROM – Patient-reported Outcome Measure.
